# Liver transplantation in children with inborn errors of metabolism: 30 years experience in NSW, Australia

**DOI:** 10.1002/jmd2.12219

**Published:** 2021-05-04

**Authors:** Noha Elserafy, Sue Thompson, Troy Dalkeith, Michael Stormon, Gordon Thomas, Albert Shun, Janine Sawyer, Shanti Balasubramanian, Kaustuv Bhattacharya, Nadia Badawi, Carolyn Ellaway

**Affiliations:** ^1^ Genetic Metabolic Disorders Service, The Children's Hospital at Westmead Sydney Children's Hospital Network Sydney New South Wales Australia; ^2^ Paediatric divison The University of Sydney Sydney New South Wales Australia; ^3^ Department of Gastroenterology, The Children's Hospital at Westmead Sydney Children's Hospital Network Sydney New South Wales Australia; ^4^ Department of Paediatric Surgery, The Children's Hospital at Westmead Sydney Children's Hospital Network Sydney New South Wales Australia; ^5^ Grace Centre for Newborn Care, The Children's Hospital at Westmead Sydney Children's Hospital Network Sydney New South Wales Australia

**Keywords:** clinical outcome, inborn errors of metabolism, liver transplantation, metabolic management pre‐ and post‐liver transplantation, organic acidemia, survival, urea cycle disorder

## Abstract

**Background:**

Inborn errors of metabolism (IEM) are a diverse group of genetic disorders that can result in significant morbidity and sometimes death. Metabolic management can be challenging and burdensome for families. Liver transplantation (LT) is increasingly being considered a treatment option for some IEMs. IEMs are now considered the second most common reason for pediatric LT.

**Aim:**

To review the data of all children with an IEM who had LT at The Children's Hospital at Westmead (CHW), NSW, Australia between January 1986 and January 2019.

**Methods:**

Retrospective data collected from the medical records and genetic files included patient demographics, family history, parental consanguinity, method of diagnosis of IEM, hospital and intensive care unit admissions, age at LT, graft type, clinical outcomes and metabolic management pre and post‐LT.

**Results:**

Twenty‐four LT were performed for 21 patients. IEM diagnoses were MSUD (n = 4), UCD (n = 8), OA (n = 6), TYR type I (n = 2) and GSD Ia (n = 1). Three patients had repeat transplants due to complications. Median age at transplant was 6.21 years (MSUD), 0.87 years (UCD), 1.64 years (OA) and 2.2 years (TYR I). Two patients died peri‐operatively early in the series, one died 3 months after successful LT due to septicemia. Eighteen LTs have been performed since 2008 in comparison to six LT prior to 2008. Dietary management was liberalized post LT for all patients.

**Conclusions:**

Referral for LT for IEMs has increased over the last 33 years, with the most referrals in the last 10 years. Early LT has resulted in improved clinical outcomes and patient survival.

**Abbreviations**
ADHD: attention deficit hyperactivity disorder
ASA: argininosuccinic aciduria
CHW: The Children's Hospital at Westmead
CPS: carbamyl phosphate synthase I deficiency
GSD: glycogen storage disease
IEM: inborn error of metabolism
LT: liver transplantation
MMA: methylmalonic acidemia
MSUD: maple syrup urine disease
NBS: newborn screening
OA: organic aciduria
OTC: ornithine transcarbamylase
PA: propionic acidemia
PEG: percutaneous endoscopic gastrostomy
PICU: pediatric intensive care unit
PLAA: plasma amino acid
TG: triglyceride
TYR: tyrosinemia
UCD: urea cycle disorder
UMS: urine metabolic screen
Urine SA: succinylacetone

## 1 INTRODUCTION

Inborn errors of metabolism (IEM) are a diverse group of disorders resulting from single gene defects which interrupt normal metabolic processes.^1^ The majority of these disorders occur either as an enzyme or transporter protein deficiency,^2^ which leads to the accumulation of toxic substrates, deficiency of the product of the enzymatic/transport reaction or failure of energy production.^3^ The cumulative incidence of IEM has been estimated to be approximately 1:1000 live births.^4^ Due to their diverse nature, clinical presentations vary from acute neonatal decompensation to later onset chronic presentations in childhood, adolescence or even adulthood.^1^

The treatment of IEM depends on three main aspects; elimination of toxic metabolites, withholding of the substrate upstream of the enzyme blockade and replenishing essential downstream products.^5^ This can be achieved by dietary modification, specific therapies to scavenge toxic metabolites and supplementation of essential products.^3^

Sometimes despite optimal management, life‐threatening metabolic decompensation continues to present serious health and neurodevelopmental risks.^6^ IEM are now the second most common reason for pediatric liver transplantation (LT).^7^ Disorders such as MSUD and UCD can be virtually cured by LT, while for other disorders such as PA and methylmalonic acidemia (MMA), LT can improve clinical outcomes.^8^

### 1.1 Aim of the study

To review the indications for and outcomes of children with IEM who underwent LT at a tertiary referral pediatric hospital.

### 1.2 Methods and patients

This retrospective study was conducted at The Children's Hospital at Westmead (CHW), Sydney, Australia, reviewed all children with an IEM who underwent LT in NSW, Australia between 1986 and 2019. One child was excluded from the study as their transplant was performed overseas. Data were collected from the medical records and genetic files and included gender, age, family history, ethnicity, parental consanguinity, method of diagnosis of IEM, number of hospital admissions, including intensive care admissions, medical and dietary management pre‐transplant, age of referral for LT, type of liver graft, clinical outcomes, diet and metabolic management post‐transplant.

## 2 RESULTS

Twenty‐one children underwent 24 LT from January 1986 to January 2019, of whom 12 were male. The youngest age at referral was 1 month (three males with ornithine transcarbamylase [OTC] deficiency) and the youngest age at LT was 5 months (one male with OTC deficiency). The lowest weight at transplant was 7.1 kg. Eighteen liver transplants were performed after 2008 in comparison to six transplants prior to 2008.

Positive family history was confirmed in five children and was considered likely in one child with a family history of early neonatal deaths of unknown cause in previous siblings born overseas. Parents of six children were consanguineous (first cousins). See Table S1 for details.

### 2.1 Clinical presentation and investigations:

Most children presented within the first week of life with an acute metabolic decompensation except:

1. Four children with peri‐natal metabolic management plans formulated following prenatal diagnosis or previous family history (one with MSUD and three with OTC deficiency). These children were investigated immediately after birth and were admitted to the PICU for prompt management.
2. Two females with OTC deficiency presented at a later age with failure to thrive, recurrent vomiting and feeding difficulties, one at 5 months of age with global developmental delay and the second at 10 months of age with developmental regression.
3. One child with MMA was asymptomatic in the newborn period. An abnormal C3 pattern was identified by newborn screening and further investigations confirmed the diagnosis.
4. Infants with TYR I presented at around 5 months of age with hepatosplenomegaly and renal tubular dysfunction.
5. One child with GSD Ia presented at 4 months of age with vomiting, failure to thrive, hypotonia and hypoglycemia.

UCD was the most common indication for LT in eight children (six children with OTC deficiency, one each with ASA and CPS1 deficiency) followed by OA in six children (four with PA and two with MMA). Other diagnoses were MSUD (n = 4), TYR 1 (n = 2) and GSD Ia (n = 1).

Seven children required immediate admission to PICU for haemofiltration after initial diagnosis of MSUD and UCD due to toxic levels of leucine or ammonia. Table 1 shows a total number of referrals that did not lead to transplantation and the mortality in the IEM specific cohort(s) without transplantation.

**TABLE 1:** Number of patients in the metabolic service and outcome

Diagnosis category (diagnosis)	Number of patients in metabolic service	Deceased (non‐transplanted)	Total number of patients referred to LT	Referrals for LT that did not proceed to transplant (were not listed)	Patients proceeded to LT	Patients deceased post‐LT
UCD (ASA, CPS, Citrin Def, CIT I, CIT II, OTC)	45	3 (OTC = 2,CPS = 1)	9	1 (OTC)	8	1 (OTC)
OA (PA/MMA)	14	1 (PA = 1)	7	1 (PA)	6	1 (PA)
AA (MSUD)	19	1	4	0	4	0
Carbohydrate Disorder (GSD Ia)	5	0	1	0	1	0

### 2.2 Genotyping

Pathogenic variants of the *OTC*, *PCCB, BCKDHB*, *G6PC* genes were identified in seven patients (three OTC, two PA one each with MSUD and GSD1a). Likely pathogenic variants were identified in the *PCCA*, *CPS1* and *OTC* genes for four patients (two PA, two CPS1, one OTC). One patient with MMA had compound heterozygous variants, one pathogenic and one likely pathogenic variant of the *MMUT* gene. One patient with OTC deficiency had a variant of uncertain significance in the *OTC* gene and a second patient had no abnormality detected. Genetic testing was not done for seven patients. See Table S1 for further detailed information.

### 2.3 Indications for and timing of referral for liver transplant

MSUD: The reasons for referral for LT included metabolic instability, difficulties with dietary management and poor patient quality of life.

UCD: Children were referred for LT at a younger age due to brittle disease with recurrent hyperammonemia and multiple hospital admissions. Males with OTC deficiency were considered for LT at an earlier age than females.

MMA: Both children with Mut° MMA had renal impairment prior to LT. One had two separate renal transplants later in life. Both patients had haemofiltration immediately prior to LT to acutely reduce plasma MMA levels and optimize metabolic control.

PA: The main indication for LT referral was to prevent further metabolic decompensation and clinical deterioration. All children had global developmental delay and seizures with some demonstrating basal ganglia abnormalities on MRI.

TYR I and GSD Ia: The risk of hepatocellular carcinoma and renal tubular dysfunction was the main indication for LT in TYR I. The child with GSD 1a had extremely poor metabolic control with a 1‐hour fasting period.

### 2.4 Biochemical parameters pre‐ and post‐LT

Peak blood ammonia and plasma leucine levels at the time of diagnosis were documented for children with UCD and MSUD, respectively. Ammonia and leucine levels normalized after LT except for one child with MSUD whose leucine remained mildly elevated. Comparing mean plasma MMA levels 3 years pre‐ and post‐LT showed significant improvements but did not normalize. Patients with PA were detected by newborn screening. Methylcitrate was not measured. Normalization of ammonia levels was achieved for all patients with PA and MMA post LT. Table 2 summrizes the Peri‐operative infusions, investigations and managment done during the process of LT.

**TABLE 2:** Peri‐operative infusions, investigations and management

	Intravenous fluids	Investigations	Medications/procedure
MSUD	Dextrose 10% + Lipids	Plasma amino acids + pre‐transplant bloods^a^	
UCD	Dextrose 10% + Lipids	Plasma amino acids + ammonia + pre‐transplant bloods^a^	Sodium benzoate + L‐arginine infusion
MMA	Dextrose 10% + Lipids	Plasma amino acids + ammonia + plasma MMA + pre‐transplant bloods^a^	Haemofiltration for 4 h prior to LT
PA	Dextrose 10% + Lipids	Plasma amino acid + acyl carnitine profile + ammonia + pre‐transplant bloods^a^	L‐carnitine infusion

^a^ Pre‐transplant bloods included full blood count, coagulation screen, urea, electrolytes, creatinine, liver function tests and blood group and cross match.

### 2.5 Type of graft

All children received grafts from deceased donors. Sixteen (66%) had split grafts, eight had whole grafts. Three children required a second LT.

### 2.6 Post‐LT survival

**FIGURE 1:**
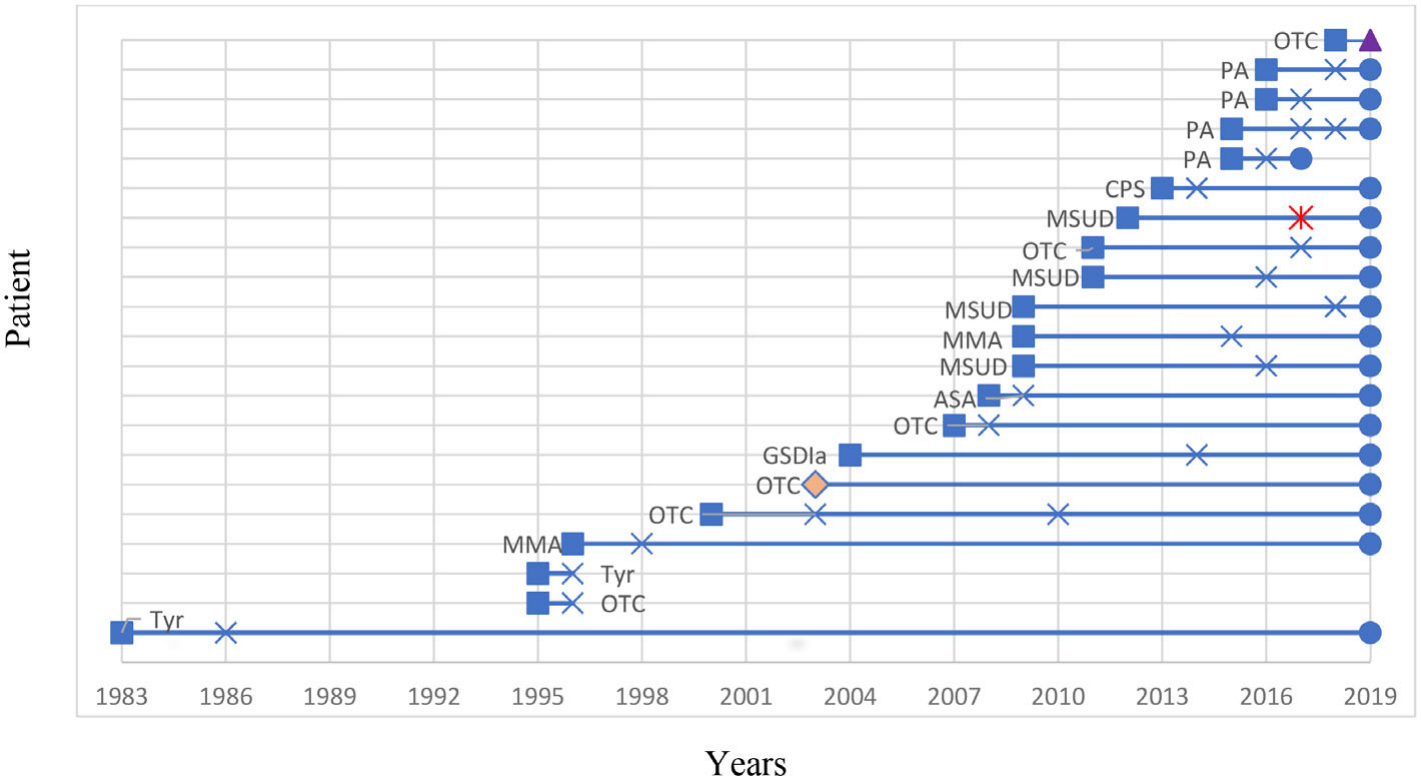
Post‐liver transplant survival. 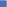, Birth year 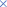 transplant year; 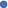, survival post‐LT; 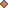, birth and transplant in the same year; 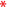, patient had two liver transplants within a short period; 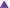, patient had LT in 2019 and still alive

Of 21 children who had LT since January 1986, three died (14%) (Figure 1). Two children died early in the series; one child with OTC deficiency died from chronic graft rejection, while one patient with TYR 1 died from peri‐operative complications (hypotension not related to blood loss during surgery). More recently one child with PA died from central line sepsis 3 months after a successful LT.

### 2.7 Hospital admissions pre‐ and post‐LT

There was decline in the mean number of hospital admissions after LT, pre‐transplant (15.4, 95% confidence interval [CI] 8.1‐22.8) to post‐transplant (8.9, 95% CI 4.8‐13.1) although this was not statistically significant (*P* = 0.1149).

### 2.8 Metabolic management pre‐ and post‐LT

Diets were substantially liberalized for all children post LT. Refer to Table 3 for further details. Prior to LT, all patients had complex restricted diets supplemented with medical formulae and foods. All patients, apart from the two patients with TYR I, had emergency unwell management plans. Gastrostomy or nasogastric feeding was used by 13 patients. Following LT, diets were liberalized to a “normal diet,” except for a mild protein restriction and simplified emergency management plans for those with PA and one patient with MSUD. The MSUD patient has had mild persistent elevations of plasma/dB leucine and allo‐isoleucine in the 3 years post‐LT (leucine mean ± SD, 297 ± 79umol/L, range 112‐481umol/L, normal range = 56‐178umol/L). Gastrostomy feeding tubes have been removed in all but two patients. Diet adequacy was qualitatively assessed for 8 of 14 patients that continue to be managed at CHW metabolic service 1 month to 15 years post LT. For the four on full oral intake (three MSUD, one OTC), all had an increased intake of natural protein over time but for three there was an inadequate intake of calcium, iron and vitamin B12, when compared to national guidelines.^9^ One patient (OTC) continued to eat a self‐restricted diet following the first LT but broadened food range almost immediately after the second LT. The others (three PA, one OTC) were reliant on gastrostomy/NG/oral feeds for some of their intake. All patients and families had improvement in their quality of life with diet liberalization.

**TABLE 3:** Metabolic dietary management pre‐ and post‐LT

	Pre liver transplant	Post‐ liver transplant
MSUD	Severe protein restriction 0.1 to 0.46 g/kg/d + MSUD medical formulae and foods	Diet normalized for three children while one remains on a modest protein restriction, 1.2 to 1.7 g/kg/d
UCD	Protein restriction 0.8 to 1.25 g/kg/d Two infants used low protein formula/foods in combination with breast milk or infant formula	Diet normalized for all
OA	Protein restriction 0.7 to 1.3 g/kg/d + MMA/PA medical formulae and foods	Diet liberalized modest protein restriction, 1.5 to 2.5 g/kg
TYR I	Protein restriction 0.5 to 0.7 g/kg/d + Phe and Tyr free medical formulae	Diet normalized
GSD Ia	Continuous/frequent PEG feeds	Diet normalized overnight feeds not required

### 2.9 Complications and outcomes

The only serious immediate post‐operative complication was acute hepatic venous outflow tract obstruction (HVOTO) in a patient with MSUD, requiring urgent re‐transplantation on day 2 using another split graft. Other vascular complications included early hepatic artery thrombosis in an OTC patient which resulted in biliary stricture and re‐transplant 7 years later. Biliary complications included anastomotic bile leaks requiring early surgical revision in two patients (one OTC, one MSUD) while another MSUD child required biliary sphincterotomy and stenting day 11 post LT for an early biliary stricture. Three patients had bowel obstructions post‐transplant (two MSUD, one PA) while a child with GSD Ia needed revision of the Roux‐en‐Y anastomosis. Acute hepatocellular rejection was successfully managed in four patients (two MSUD, two OTC) while one child with OTC died from chronic rejection in the early era of the transplant program. One child with PA had re‐transplantation after 8 months due to chronic graft rejection and severe pruritis. In summary, three children required re‐transplantation and three died post LT.

Due to the retrospective nature of this study, developmental assessments were not available for all children. Data available post LT showed that children with MSUD had mild to moderate intellectual impairment, ADHD and generalized anxiety. Children with UCD had relatively better developmental outcomes with normal intellect except for two children with mild/moderate intellectual disability. One infant sustained an acquired brain injury after birth due to severe hyperammonemia and has profound intellectual impairment and global developmental delay. The children in our cohort with PA have global developmental delay. One child with PA suffered a metabolic stroke prior to LT. These children continued to make developmental gains post LT. To date, no patients have had a metabolic stroke or cardiomyopathy post LT. The two with MMA have normal development and intellect.

## 3 DISCUSSION

LT has emerged as an effective treatment modality for some people with an IEM.^10^ With improved surgical techniques and pre and post‐transplant management, survival has improved significantly.^6^ Graft and patient survival post LT requires lifelong, safe and effective immunosuppression, which balances adequate immunosuppression to prevent graft rejection, yet avoiding complications of excessive immunosuppression such as recurrent infections and malignancies.^11^ Since most IEM result from a specific enzyme defect with a structurally normal liver, LT has become a unique form of enzyme replacement therapy.^12^

Children with IEM are generally prioritized on LT waiting lists due to the risk of sudden life‐threatening metabolic decompensation.^13^ In our study, all the children received grafts from deceased donors, two thirds of which were split grafts including one used for re‐transplant. Compared with whole LT, split grafts reduce waiting times for recipients. Equal graft survival rates have been described due to careful selection of donors and surgical team experience.^14^ The availability of donors as well as preference for deceased or living‐related donors varies between major LT centers. The usual practice in our center is to reserve the use of living‐related donors for graft failure or emergency situations. This is in comparison to other centers where living‐related donors are the first choice due to the scarcity of deceased donors. In a study conducted by Kasahara et al, analyzing the Japanese multicenter registry, there was no significant difference in survival using deceased donors.^15^ In Australia, access to deceased organs has improved and all of our patients received carefully selected deceased donor organs. Domino LT is not performed in our center, largely due to logistic difficulties and organ availability.

Current guidelines recommend aggressive medical therapy for neonatal onset UCD and LT as early as 3 months of age and once the patient's weight is over 5 kg. To demonstrate the importance of early referral and transplant for these patients, Perito et al reported six patients who died under 2 years of age at the time of listing while waiting for LT (median waiting list time 36 days).^13^ This emphasizes the importance of utilizing living‐related donors if required in emergency situations.

Due to the retrospective nature of our study, complete neurological data were not available, but early LT has demonstrated improved long‐term neurological outcomes.^16^ In our cohort, diets were liberalized and ammonia scavengers medications were not required after LT. This favorable outcome has been observed in other studies.^17^

High mortality rates are reported for patients with OA even with early diagnosis and aggressive medical management, while survivors can have severe neurological complications.^18^ LT is generally recommended in early childhood to prevent morbidity from recurrent metabolic decompensation.^13^ Varying levels of developmental delay and neurological deficits are usually present and in keeping with our findings.^19^ Patients with OA who had LT usually demonstrate improved metabolic stability, diet liberalization and better quality of life. Metabolic stroke, cardiomyopathy and renal disease can occur even after a successful LT.^8^ In our cohort diets were liberalized cautiously and were individualized for each patient. A similar approach was noted in two studies conducted analyzing the data of patients with OA who underwent LT.^20^, ^21^

Renal transplantation was required for one of our two MMA children. The current transplant approach for MMA patients is to offer LT alone, combined liver and kidney transplant or kidney transplant alone according to the patient's clinical status. A kidney transplant is often required as well as LT due to the high expression of the methylmalonyl CoA mutase activity in the kidney and MMA‐associated renal dysfunction.^22^ Hemofiltration was performed for both children immediately prior to LT. The use of pre‐transplant hemofiltration needs careful consideration. Kamei K et al, reported pre‐transplant hemofiltration led to reduced plasma MMA levels but some patients had severe lactic acidosis and one patient died.^23^

There is currently no consensus on when is the best age to offer LT for patients with MSUD.^24^ In our study, children were transplanted at a median age of 6.21 years, which is similar to data reported by Perito et al.^13^ Diaz VM et al followed up eight patients with MSUD who had LT at a younger age (mean of 1.78 years, range 1.5‐2.5 years). No further metabolic decompensation occurred in survivors and post‐LT diets were normal.^25^ This is similar to the results of our study. Small elevations but more stable plasma leucine levels with increased diet protein intake post‐LT were also reported by Mazariegos et al, and mild plasma leucine levels were observed with intercurrent illnesses.^24^ Almost complete normalization of the diet was achieved in our MSUD cohort and gastrostomy feeding was no longer required. It might be expected that children who had been on very constrained diets since early infancy would struggle to introduce a range of new foods. This does not appear to have been the case for this cohort, but intake of higher protein foods was limited risking inadequate intake of iron, vitamin B12 and calcium. Dietetic follow‐up post LT for the full cohort has been limited, but ongoing review of feeding behaviors and nutritional adequacy appears appropriate.

The two children with TYR I, had transplants very early in the series (1986 and 1996) when phenylalanine and tyrosine restricted diets were the only treatment options. In 1992, 2‐(2‐nitro‐4‐trifluoromethylbenzoyl)‐1, 3‐cyclohexanedione (NTBC) was introduced as a novel treatment, and the need for LT dramatically decreased.^6^

While LT has become standard care for some IEM, approximately 40% of patients will experience complications. In this series, we describe several complications, including three (14%) re‐transplants and three (14%) deaths. Vascular complications include hepatic artery patency, the main determinant of a successful outcome. Other complications include acute or chronic graft rejection, biliary stricture, bile leak and malignancy including post‐transplant lymphoproliferative disorder.^26^, ^27^

Proceeding to LT requires careful consideration by the multidisciplinary team caring for children with IEMs in consultation with the patient and/or patient's family. Parents may face many challenges and stressors including financial and employment pressures, parental and family distress and relationship disruptions. Anxiety and depression can be experienced while waiting list for a LT and behavioral problems can be exacerbated. Appropriate counseling and support should be available to ensure optimal medical and psychosocial outcomes.^28^

## 4 CONCLUSIONS

Advances in surgical techniques, organ retrieval and preservation, immune suppression and pediatric intensive and medical care, have led to improved long‐term outcomes for children with IEMs undergoing LT. We have shown increased numbers of referrals for LT over the last 33 years, particularly in the last decade. LT is considered an effective treatment for patients with some IEMs.

## CONFLICT OF INTEREST

Noha Elserafy, Sue Thompson, Michael Stormon, Gordon Thomas, Albert Shun^,^ Janine Sawyer, Shanti Balasubramaniam, Toy Dalkeith, Kaustuv Bhattacharya, Nadia Badawi and Carolyn Ellaway declare that they have no conflict of interest.

## AUTHOR CONTRIBUTIONS

Noha Elserafy is the principal investigator, she contributed to the study design, data collection, analysis and drafting the manuscript. She also participated in the pertinent aspect of this project planning. She serves as the guarantor for the article. Sue Thompson participated in the study design, data collection and planning. She contributed to writing and revising the manuscript. Troy Dalkeith contributed data collection, writing and revising the manuscript. Michael Stormon contributed to interpretation of data, writing and revising the manuscript. Gordon Thomas contributed to interpretation of data, writing and revising the manuscript. Albert Shun contributed to interpretation of data, writing and revising the manuscript. Janine Sawyer contributed ton revising the manuscript. Shanti Balasubramaniam contributed patients to this cohort and reviewed the final version of the manuscript. Kaustuv Bhattacharya participated in the study design and planning and revising the manuscript. Nadia Badawi principle supervisor, participated in the study design, planning, revising the manuscript. Carolyn Ellaway principle supervisor, participated in the study design, planning, data analysis and writing and revising the manuscript.

## ETHICS STATEMENT

This project was approved by the Sydney Children's Hospitals Network Human Research Ethics Committee's Executive Committee on the April 10, 2018. HREC Reference: LNR/18/SCHN/12. All patients' details were de‐identified and kept confidential. A waiver of consent was approved by the SCHN Ethics Committee. This project is compliant with the Helsinki Declaration, and the National Statement on Ethical Conduct in Human Research https://www.nhmrc.gov.au/about-us/publications/national-statement-ethical-conduct-human-research-2007-updated-2018#toc__296. This project contains no studies on animals.

## Supporting information


**Table S1.** Supporting information.
